# Perioperative Nursing as the Guiding Thread of a Prehabilitation Program

**DOI:** 10.3390/cancers14215376

**Published:** 2022-10-31

**Authors:** Fernando Dana, Raquel Sebio-García, Beatriz Tena, Marina Sisó, Francisco Vega, Amaia Peláez, David Capitán, Marta Ubré, Ana Costas-Carrera, Graciela Martínez-Pallí

**Affiliations:** 1 Anesthesiology Department, Hospital Clínic de Barcelona, 08036 Barcelona, Spain; 2 Institut d’Investigacions Biomèdiques August Pi i Sunyer, Universitat de Barcelona, 08007 Barcelona, Spain; 3 Physical Medicine and Rehabilitation Department, Hospital Clínic de Barcelona, 08036 Barcelona, Spain; 4 Department of Endocrinology and Metabolic Diseases, Hospital Clinic de Barcelona, 08036 Barcelona, Spain; 5 Psychiatry Service, Hospital Universitari Central de Asturias, 33011 Oviedo, Spain; 6 Biomedical Research Networking Center on Respiratory Diseases (CIBERES), 28029 Madrid, Spain

**Keywords:** prehabilitation, oncology, perioperative nurse, case manager

## Abstract

**Simple Summary:**

This article describes the process of creating a prehabilitation unit in our center and the role of perioperative nursing. Initially, the project was launched with the performance of a research study on prehabilitation for gastrointestinal cancer surgery. The results of this study encouraged us to continue the implementation of the unit. Progressively, multimodal prehabilitation programs focused on each type of patient and surgery were developed. Currently, our prehabilitation unit is a care unit that has its own gym, which allows supervised training of cancer patients prior to surgery. Likewise, the evolution of perioperative nursing in the unit is described: from collaboration and assistance in the integral evaluation of the patient at the beginning to current work as a case manager; a task that has proven extremely important for the comprehensive and continuous care of the patient.

**Abstract:**

Multimodal preoperative prehabilitation has been shown to be effective in improving the functional capacity of cancer patients, reducing postoperative complications and the length of hospital and ICU stay after surgery. The availability of prehabilitation units that gather all the professionals involved in patient care facilitates the development of integrated and patient-centered multimodal prehabilitation programs, as well as patient adherence. This article describes the process of creating a prehabilitation unit in our center and the role of perioperative nursing. Initially, the project was launched with the performance of a research study on prehabilitation for gastrointestinal cancer surgery. The results of this study encouraged us to continue the implementation of the unit. Progressively, multimodal prehabilitation programs focusing on each type of patient and surgery were developed. Currently, our prehabilitation unit is a care unit that has its own gym, which allows supervised training of cancer patients prior to surgery. Likewise, the evolution of perioperative nursing in the unit is described: from collaboration and assistance in the integral evaluation of the patient at the beginning to current work as a case manager; a task that has proven extremely important for the comprehensive and continuous care of the patient.

## 1. Introduction

Despite advances in surgery safety, postoperative complications continue to have a marked negative impact on clinical outcomes, quality of life, and economic costs [1,2,3]. Currently, sedentary lifestyle and frailty are recognized as determining factors of surgical prognosis [4,5]. The prevalence of both traits clearly increases with age, but it is also associated with chronic diseases that result in a progressive deterioration of the individual’s cardiorespiratory reserve and functional capacity [6]. Preoperative identification of those patients with high surgical risk aims to establish strategies for the prevention of potential complications, with the intention of reducing their incidence and severity [7,8,9]. Increasing evidence on the potential of prehabilitation to optimize physical and psychological resilience to cope with the stress generated by major oncological surgical procedures has been generated over the last few years.

Prehabilitation programs are patient-tailored, typically multimodal, and address several aspects for improving risk factors. The combination of exercise training together with other measures such as optimization of nutritional status, especially in cancer patients, and psychological support [10] are proposed as strategies with a significant positive impact on the outcome of the surgical patient [11,12]. While evidence-based benefits of multimodal prehabilitation have been demonstrated in highly controlled settings, the true challenge remains its implementation in a real-world scenario and the successful transferability of the results across heterogeneous sites [13].

Despite this growing evidence, prehabilitation has not yet become part of standard referral practices by surgeons at most centers. Prehabilitation programs should be designed from a multidimensional approach taking into account all stakeholders involved (healthcare professionals, patients, and providers). An implementation research approach, combining experience-based co-design and quality improvement methodologies, was recently conducted at Hospital Clinic de Barcelona [14]. As part of the co-creation process, we undertook two design-thinking events (October–November 2017 and June 2021) to generate a work plan fostering service scalability with a population-based orientation. The implementation process was assessed using the Comprehensive Framework for Implementation Research (CFIR), leading to the identification of key performance indicators for long-term service follow-up after adoption that were reported using the Avedis–Donabedian approach.

The aim of the current report is to summarize from a more pragmatic point of view the process of implementing a prehabilitation unit as an integrated care program at the Hospital Clinic of Barcelona (HCB), emphasizing the evolution of the role of perioperative nursing, from its beginnings as an expert aid to the anesthesiologist until its consolidation as a case manager at the present time.

## 2. Prehab Unit Implementation Process

Prehabilitation is a complex process that requires a multidisciplinary team, proper facilities, and adequate equipment. To be efficient and adopted by individual patients, an adequate design of patient workflow through the perioperative pathway, where many professionals are involved, is crucial. Hence, the deployment of prehabilitation in a real-world scenario involves several logistic challenges.

After almost a decade from our first study, the prehabilitation service at HCB currently receives more than 200 high-risk surgical referrals per year from different surgical specialties. Patients are referred by the surgeon or the anesthesiologist after the preoperative assessment. The intervention is carried out by a multidisciplinary team led by the Anesthesiology Department combining hospital and community-based actions. The phases of the implementation process of the unit are described below. The unit is fully focused on the high-risk surgical patient, who is empowered to participate in his/her surgical process in order to achieve the best possible results. The sense of accompaniment by the members of the unit during this stressful process is also a key aspect for the success of the intervention [15].

Throughout the years, the demands of an increasingly complex healthcare system have meant that the contribution of nurses has undergone a process of redefinition. Whereas in the past nurses were not considered integral members of a clinical care team (despite their responsibilities), today they have much more autonomy and recognition, enjoying an increasingly collaborative relationship with physicians and other healthcare professionals. In our opinion, nurses may play a key role in delivering appropriate and coordinated care interventions before surgery, to prevent postoperative complications and enhance surgical recovery. Prehabilitation is based on the concept: everyone working together as a team caring for the preoperative optimization of the patient. The nurse would act as a trusted first point of contact to reassure patients and link them with the rest of the team.

## 3. Experimental Phase (2013–2016)

Generating solid evidence was our first step. We began with a three-year (2013–2016) double-blind RCT that evaluated the efficacy of prehabilitation in high-risk patients undergoing major gastrointestinal surgery. Patients at high risk for postoperative complications were specifically included in the study [12]. The prehabilitation program core was physical training, including three main aspects: (i) a motivational interview, (ii) a high-intensity resistance exercise training program in the hospital, and (iii) the promotion of physical activity. After completing a 6-week program, prehabilitation proved to be a protective factor for both the number of postoperative complications and the risk of presenting more than one complication. The study also showed that prehabilitation reduced the length of stay in the intensive care unit [12]. The cost-effectiveness analysis showed that the intervention could generate health value: prehabilitation added costs to health care, but these were compensated with a reduction in postoperative complications and a shorter hospital stay. During these initial years, in addition to demonstrating the efficacy of prehabilitation, we had the opportunity of preparing patient information and solve ethical issues, and to search and adapt local structures and support systems (data acquisition), thus gaining experience for the clinical translation. The involvement of passionate professionals, “prehabilitation believers”, was also crucial for the adoption process.

In this phase, the role of perioperative nursing was to collaborate supporting the team for the initial assessment of the patients, as well as in the pre-surgery re-evaluation. Once visited by the anesthesiologist, the tasks performed included assisting the patients to fill in the different questionnaires predetermined in the study as well as collaborating in performing physical functioning tests such as the 6-Minute Walk Test (6MWT) the 30” Sit-to-Stand Test or the hand-grip strength. These tests were repeated in the pre-surgery evaluation of the patients. In addition, nurses were also involved in performing administrative assistant tasks such as receiving and registering patients in the hospital’s local system.

## 4. Pilot Phase (“Low-Cost” Unit): 2016–2017

Following these encouraging results, the prehabilitation service was deemed ready for implementation as a mainstream service at HCB, leading to the creation of the Prehabilitation Unit in 2016. During the initial 12 months period, the main purpose was to demonstrate the feasibility of a real-world prehabilitation program in a tertiary care hospital, and to generate the service workflow design of the intervention. Due to limited resources and program capacity, patients with lower baseline functional reserve were prioritized since they were more likely to benefit from prehabilitation [16].

A preoperative schedule that allowed at least 4 weeks of prehabilitation was designed (Table 1). In addition to gastrointestinal surgeries, we progressively included patients from other surgical specialties (i.e., cardiac, thoracic). The program adhered to the ERAS (enhanced recovery after surgery) [17] recommendations established for the different surgeries. At this point, given the growing evidence that nutritional counseling and supplementation as well as psychological support may improve the results, we also initiated a multimodal approach including both interventions in our unit [18].

Patients initiated the program after the preanesthetic visit. On the first day, a holistic characterization of the patients was carried out as patients were introduced to the multidisciplinary team and the three pillars of the program. Identification of relevant clinical features and comorbidities [19,20], assessment of nutritional and psychological status, and assessment of basal aerobic capacity and physical activity levels were performed to personalize the program. Factors that would affect their access (social, logistical, etc.) and adherence to the physical activity program were also investigated.

At that time, due to the absence of specific facilities for supervised training, the program only included home-based physical training and a personalized promotion of physical activity plan designed after a motivational interview with the physiotherapist. Replacing sedentary time with physical activity by increasing the amount of daily movement in short sessions is a well-known strategy to improve health, especially in previously inactive individuals [21]. Specifically, the physical activity program consisted of increasing the number of daily steps, measured with a pedometer, and/or optimizing the intensity of the walk, measured with the modified Borg scale [22,23].

**Table 1:** The most important summaries of the trimodal prehabilitation program in the pilot phase.

Inclusion criteria	High-risk surgical candidates for postoperative complications, defined by age > 70 years and/or ASA class III-IV (scheduled for major digestive, cardiac, thoracic, gynecologic, or urologic surgeries) and/or patients suffering from severe deterioration caused by cancer and undergoing very aggressive procedures such as esophagectomy, total gastrectomy, cystectomy, or gynecological oncological surgery.
Functional capacity assessment	Six-minutes walking test (6MWT) [24]. 30” Sit-to-Stand Test [25]. Hand grip strength [26]. Fitness assessment with the Duke Activity Status Index (DASI) [27]. Physical activity level: Yale Physical Activity Survey (YPAS) [28,29] questionnaire, short version.
Physical activity	-Included home-based physical training and a personalized promotion of physical activity plan designed after a motivational interview with the physiotherapist. -Physical activity promotion through number of daily steps, measured with a pedometer. -At least two resistance training sessions per week through body-weight exercises or using home-available materials (i.e., water bottles).
Follow up	-A weekly follow-up visit was made in the unit to review physical activity goals. -Pedometer to encourage them to achieve the daily step goal so physical therapists could assess improvement. -Patients participated in a 1 h group fitness class led by a physical therapist, and were monitored for symptoms contraindicating exercise.
Nutritionist	Based on their initial assessment, patients received recommendations for a healthy, balanced diet or a diet tailored to their digestive symptoms. Group sessions, except for patients with special nutritional requirements (for example, esophagectomies) in which individual sessions were provided. Patients with high risk of malnutrition (Malnutrition Universal Screening Tool ≥ 2) [30] were treated by a qualified dietician.
Psychology	This intervention was recommended especially in patients who presented signs of anxiety or depression (defined as a score on the Hospital Anxiety and Depression Scale (HADS) > 8) [31]. The group-based sessions of ninety minutes each included breathing and relaxation exercises led by a psychiatrist or psychologist with expertise in mindfulness.

The implementation of a multidisciplinary unit made the flow of patients somewhat more complicated. On a typical day in the unit, patients who came for the first assessment mixed with those who had follow-up visits with one or several professionals, in addition to those who came for their pre-surgery evaluation. Nurses began to perform other tasks such as the coordination and scheduling of all activities, accompanying and guiding the patients during the program, and informing patients and caregivers about the surgical process.

## 5. Inauguration of the Gym, Redefining the Program (2017–2019)

### 5.1. Patient Journey in the Prehabilitation Unit

In 2017, the availability of an appropriate space for a hospital gym made it possible to consolidate a supervised training program in this second period. Endurance exercise training is a key component of prehabilitation as it is the best approach to improve aerobic capacity. Particularly, high-intensity training can be more effective than moderate-intensity exercise training or an unsupervised home exercise program when limited time is available to achieve changes in oxygen consumption [32,33].

**Figure 1:**
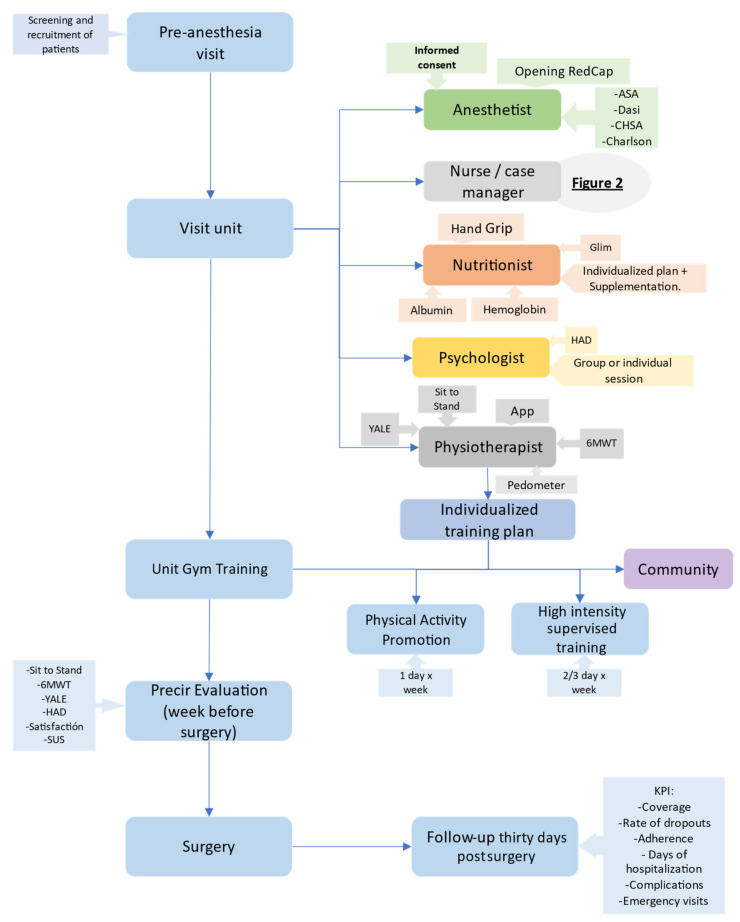
General circuit of the PreHab unit.

As an integrated care service (Figure 1), there was a clear need for refinement of the standard prehabilitation intervention to increase healthcare efficiencies. The prehabilitation program was defined as a multimodal program based on these fundamental pillars:

(1) Personalization of an aerobic and resistance exercise program, respiratory incentive, and promotion of physical activity. Therefore, two modalities of the program were developed with physiotherapists deciding which is more appropriate for each patient The programs currently available are:

- Supervised training: This modality includes 45 min sessions of high-intensity supervised interval training with a stationary cycle ergometer or treadmill (Technogym^®^ Excite Bike; Cesena; Italy) and muscle strength training (Technogym^®^ Plurima Multistation Wall; Cesena; Italy) two or three days a week under the supervision of the physical therapist in the unit’s gym. This program is prioritized for the most deconditioned patients and for the most aggressive surgeries.
- Promotion of physical activity (PPA): Patients who for different reasons cannot attend the supervised training program (difficulties in transportation, unwillingness to attend supervised sessions, etc.) are referred to this modality, which includes a weekly session supervised by the physiotherapist to reinforce instructions, assess progress, and review goals and two or three non-supervised sessions at home or local gyms.

The exercise training is performed on top of encouragement of daily physical activity through fitness trackers or pedometers.

(2) Optimization of nutritional status. When individuals increase their level of physical activity, they need an adequate protein substrate to cope with the gain in muscle mass during this period. In addition, certain pathologies condition an inadequate intake and the surgery itself frequently determines prolonged periods of fasting. The MUST (Malnutrition Universal Screening Tool) has been used as a nutritional screening tool. In addition, the GLIM (Global Leadership Initiative on Malnutrition) [34] has been proposed as useful to diagnose malnutrition.

The nutritional intervention is managed by a registered dietitian. Individualized diet therapy is recommended, and nutritional supplementation is prescribed if it is necessary.

In addition, those patients with documented iron loss or with gastrointestinal cancer and anemia (Hb < 12 g/dL) are treated with intravenous iron.

(3) Psychological intervention. One weekly 2 h session of mindfulness is organized, and open to all patients who wish to attend upon request. In the unit, there is also a reference psychologist who can visit patients individually depending on patient needs.

### 5.2. Consolidation of the Prehabilitation Program and COVID-19 Outbreak (2019–2021)

Since 2019, the prehabilitation program has been complemented by a digital solution (https://eithealth.eu/product-service/paprika, accessed on 30 July 2022) developed by the prehab team. The team conceptualized the need for covering: (i) patients’ accessibility and empowerment; (ii) enhanced management of care paths; and (iii) collaborative work between two or more stakeholders (patient/caregivers and professionals), eventually from different healthcare tiers/providers. The application tracks patients’ daily steps, sends motivational messages, and provides access to educational material in the three main pillars (nutrition, physical activity, and psychological support) [35].

The duration of the prehabilitation program is mainly determined by the waiting time until surgery. At the end of the program, a re-evaluation of the patient’s functional, nutritional, and psychological status is performed before surgery, in order to monitor the effects of our intervention. At the same time, patients are asked to complete a satisfaction survey to evaluate their experience with the care provided, and the SUS (System Usability Scale for the Assessment of Electronic Tools) [36] questionnaire to assess the performance of our mobile application. Some key performance indicators (KPIs) have been defined in order to carry out a long-term evaluation of the structure [13], processes, and results of the prehabilitation program in clinical practice. The analysis of all these data provides valuable information on the safety, quality, and efficacy of the intervention.

Since our unit is targeted to high-risk patients, especially those with chronic complex conditions, it is necessary to provide more joined-up care (continuum of care). Typically, such patients are under the care of multiple therapists (e.g., cancer specialist, endocrinologist, cardiologist, and hospital specialists) as well as the broader health and social care apparatus (e.g., hospital, hospice, GP, social worker, community nurse, and physiotherapist). Therefore, this complexity of closely collaborating multilevel caregivers and their patients needs a professional to act as a “common thread” dedicated to ensuring that the program provides the maximum preventive effect in an efficient manner. Good coordination among all individuals involved in the program would result in better patient experience, better care team cooperation, and fewer unplanned events.

**Figure 2:**
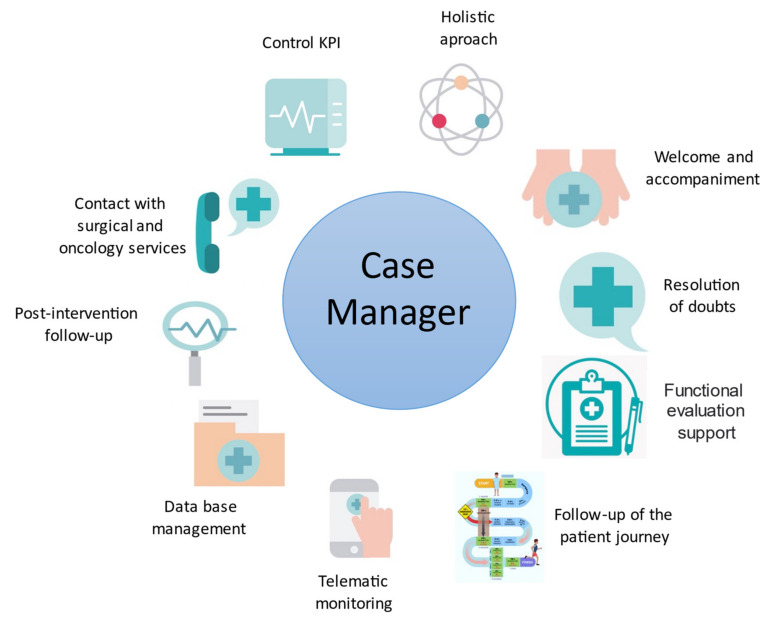
Case manager tasks in the Prehabilitation Unit.

Therefore, the role of nursing evolved in this phase from collaborating in the evaluation of patients and accompanying them during visits, to carrying out case management tasks. Case management is defined by the Case Management Society of America (CMSA) as “a collaborative process of assessment, planning, facilitation, care coordination, evaluation and advocacy for options and services to meet an individual’s and family’s comprehensive health needs through communication and available resources to promote patient safety, quality of care, and cost-effective outcomes” [37,38,39,40,41,42]. The main objective of case management in prehabilitation is to achieve continuity in health care by integrating all the care processes and thus ensuring that the patient is accompanied throughout the whole surgical process. Prehabilitation nurses continued to carry out the task of accompanying patients and their families during their visits to the unit. It included the reception, the optimization of the flow of visits of the different professionals, and the reinforcement of the information in order to solve doubts, not only about the program, but also about any step of the surgical process. They also act as a link between the different professionals, arranging periodic contact with the different members of the care team to update the situation and status of each patient within the program (Figure 2).

### 5.3. Prehabilitation Tasks of the Nurse/Case Manager

Patients’ follow-up is also performed by the prehabilitation nurses (Figure 2). They make sure that patients attend all the scheduled visits to the unit, detect patients who are drop-outs and the reasons, and are in contact with the surgical and oncology services to immediately know about the patients’ clinical evolution and dates of surgery. When the date of surgery is set, they schedule the appointment for the pre-surgery evaluation. During the pre-surgery evaluation, they ensure the patients receive the satisfaction questionnaire of the program. They also promote telematic follow-up through the app, sending messages to assess patients’ status, answering questions, and accompanying them during the program, with special emphasis on patients who do not go to the gym regularly. They collaborate in the follow-up of the patients during the 30 postoperative days after surgery to assess the duration of hospital stay, any surgical or medical complications, or readmissions.

Another important task performed by nurses is the preparation, updating, and monitoring of the unit’s database (Redcap^®^) [43,44], in order to check that all the key components of the program are registered, which facilitates patient monitoring by all the healthcare professionals during the program. An inclusion and final report are created and uploaded into the hospital system when a patient is included in the program and when this is finished, respectively. The nurses ensure all this information is immediately recorded in the patient’s medical history and is available to be consulted.

## 6. Next Steps: Community Program (2021–Ongoing)

Currently, the multimodal prehabilitation program has been consolidated at HCB as a standard service for approximately 250 candidates per year undergoing major surgeries in different specialties. It is of note that the capacity of the prehabilitation unit covers less than 20% of the estimated demand, mainly due to the limited capacity of the exercise training facilities at HCB. This aspect, together with patients’ logistic/accessibility limitations, prompted a pilot project offering physical training for selected patients in the community. The monitoring of physical activity is carried out through our digital solution if patients have a smartphone, or by telephone otherwise. In addition to the explained tasks, nurses have an important role in patients’ remote monitoring in this phase. As such, they act as the reference person for patients within the prehabilitation team so that patients can discuss any doubt regarding the program or the surgical process either by the app or by phone, and prehabilitation nurses can solve these questions themselves or refer them to the specific professional if necessary.

Prehabilitation nurses are a key part of the team since they maintain the continuity of care, ensure the proper use of resources, and accompany patients during the whole process, acting as a link between them and the rest of the team. Their tasks have been extended (Table 2) parallel to the growth of the prehabilitation unit becoming case managers. Currently, they also participate in the periodic control of the KPIs of the unit, and the redesign of care practice, when necessary, as well as in the different research studies that are underway.

## 7. Conclusions

The creation process and evolution of a prehabilitation unit in a tertiary hospital have been described. The initial project evolved from a research study to a multidisciplinary prehabilitation unit for high-risk patients scheduled for different types of major surgical procedures. Currently, the unit provides assistance to a large number of patients, most of them frail patients with high comorbidity. The role of perioperative nursing has also changed parallel to the development of the unit, with nurses becoming mainly case managers with tasks involving patient accompaniment, assessment, and coordination, in order to improve patient care.

## Figures and Tables

**Table 2:** Evolution task: nurse/case manager.

Accompaniment of patients	Providing emotional, intellectual, and psychological support. Work with the multidisciplinary health care team to measure the effectiveness of the case management plan and to monitor outcomes. Coordinate a patient’s care.
Information	To patients and caregivers. To professionals. Plan, follow-up, and data of surgery.
Reporting	Monitoring of patient care and keeping of records. Contact with the surgical and oncology services.
Communication	Identification of patient’s problems and then communicate these verbally or in writing to other members of the health team. Through the APP or by telephone.
Teaching	Prepare the patient for the post-operative.
Testing support	6MWT. Questionnaires. STS.
Research	Studies that are underway. Presentation at conferences.

6MWT: six minutes walking tests, STS: sit to stand.

## Data Availability

Not applicable.
